# Surgical Resection of Cardiac Paragangliomas Surrounding Both Coronary Arteries

**DOI:** 10.1016/j.cjco.2023.10.006

**Published:** 2023-10-13

**Authors:** Rohit K. Kharbanda, Roemer J. Vos, Hans Morreau, Jerry Braun

**Affiliations:** a Department of Cardiothoracic Surgery, Leiden University Medical Center, Leiden, The Netherlands; b Department of Pathology, Leiden University Medical Center, Leiden, The Netherlands

**Paragangliomas are neuroendocrine tumours that are often highly vascularized and, in most cases, benign. The gold-standard treatment is surgical resection for various reasons; however, this can be challenging because of close proximity to coronary arteries. Paucity of data has resulted in different (hybrid) treatment strategies with unknown long-term outcomes, especially with respect to coronary reintervention after stenting. Here, we report a unique patient with multiple cardiac paragangliomas adjacent to both coronary arteries who underwent complete surgical resection. Cardioplegic arrest is recommended to facilitate resection and, if needed, to perform concomitant coronary artery bypass grafting or reconstruction of cardiovascular structures.**

Paragangliomas are neuroendocrine tumours that are often highly vascularized and, in most cases, benign. Approximately 2% of all paragangliomas are localized in the chest, of which cardiac paragangliomas are the most uncommon.^1^^,^^2^ Metabolically active cardiac paragangliomas may result in symptoms of catecholamine excess, of which angina pectoris is the most hazardous. Also, local growth may cause compression of cardiovascular structures, and there is a (low) risk of malignancy. The gold standard therefore is complete surgical resection, which can be challenging because of close proximity to coronary arteries. Paucity of data has resulted in different (hybrid) treatment strategies with unknown long-term outcomes.^1^^,^^3^ We present a case of a patient with multiple cardiac paragangliomas adjacent to both the right and left coronary arteries who underwent direct complete surgical resection.

## Case

**Figure 1:**
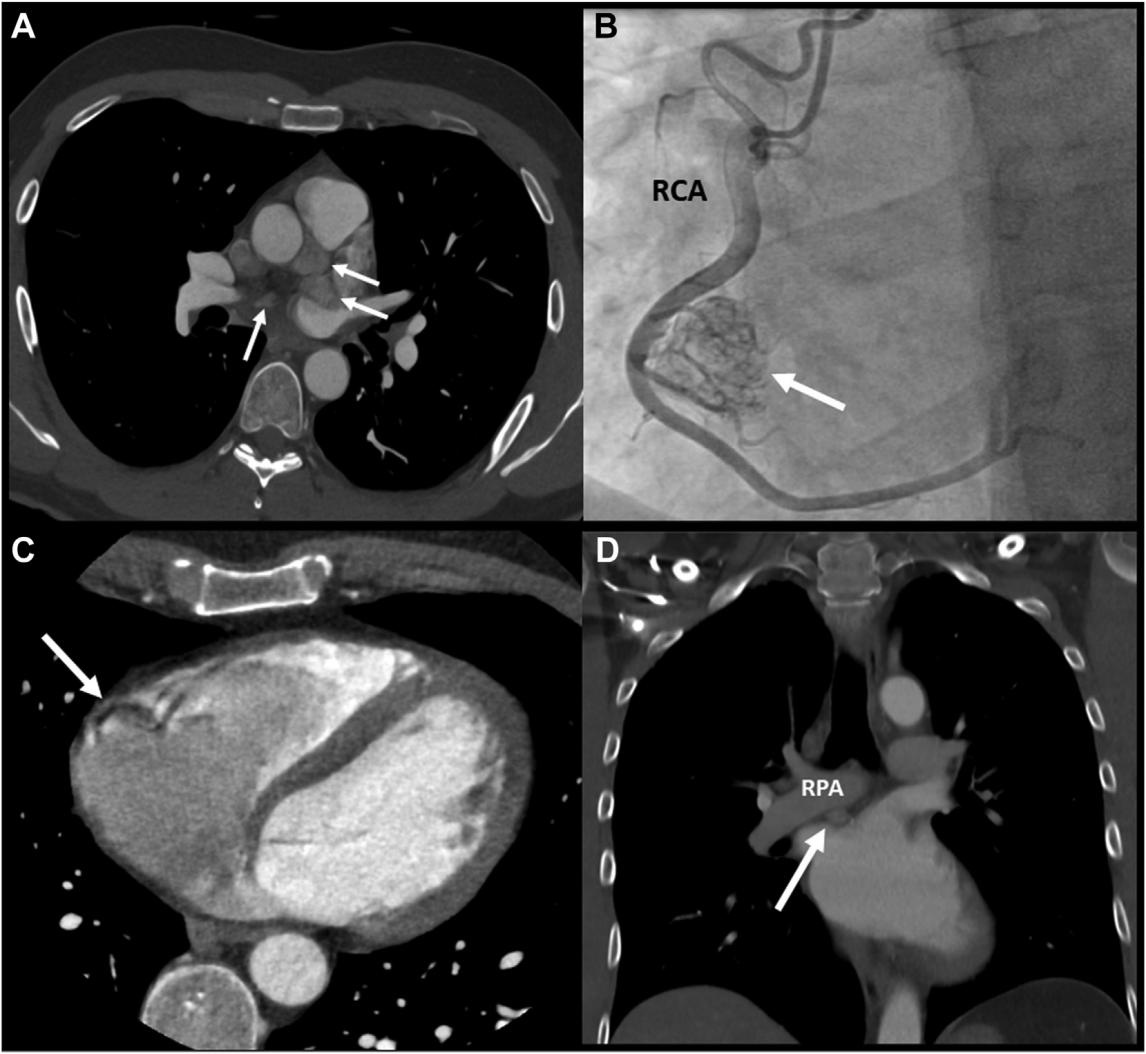
Preoperative imaging of the cardiac paragangliomas, which are indicated with a **white arrows**. (**A**) Transverse view of computed tomography (CT) scan demonstrating multiple paragangliomas with **white arrows**. (**B**) Angiogram of the RCA demonstrating the vascularization of the paranglioma adjacent to the RCA. (**C**) Transverse view of CT scan showing the paraganglioma near the RCA. (**D**) Frontal view of CT scan showing the paraganglioma between the RPA and the left atrium. LCA, left coronary artery; RCA, right coronary artery; RPA, right pulmonary artery.

A 43-year-old male patient, with a succinate dehydrogenase complex subunit D mutation and a history of nonsecreting paragangliomas in the neck (watchful waiting) and extra-adrenal paragangliomas (surgical resection), underwent yearly follow-up chest computed tomography (CT) scan. Ten years after initial diagnosis, multiple cardiac paragangliomas were observed. As shown in Figure 1, A-D, 4 paragangliomas were localized: near the left main coronary artery (2 cm × 2 cm); in the right atrioventricular groove (2.5 cm × 1.5 cm, Fig. 1, B-C); at the roof of the left atrium (2 cm × 2 cm); and between the right pulmonary artery and the left atrium (1 cm × 1 cm, Fig. 1D). Feeding collaterals from the right coronary artery were visualized during coronary angiography (Fig. 1B).

The patient was discussed in a multidisciplinary team. Although the patient was asymptomatic, and the lesions were nonsecretory, surgical resection was preferred because of the critical localization of the paragangliomas near the left main and right coronary artery. After shared decision making, the patient was scheduled for surgical resection 6 months after diagnosis. Five days before surgery, doxazosin (8 mg twice a day) was prescribed to prevent intraoperative hypertension.

Because of the close relation of the tumours with the coronary arteries, and possible invasiveness in the atrial wall, the decision was made to operate on cardiac arrest. After median sternotomy, normothermic cardiopulmonary bypass was initiated, and the heart was arrested using cardioplegia. Bicaval venous cannulation is preferred in cases with possible invasiveness of the tumours in cardiac tissue. To expose the paragangliomas near the left atrium and the right pulmonary artery, the aorta was transversally opened after dissecting the aorta from the main pulmonary artery. Both cardiac paragangliomas infiltrated the left atrial wall. Small feeding arterial branches from the coronary arteries were clipped. Sharp, diathermic, and blunt dissection was used to reach and completely excise the paragangliomas. Complete resection, including atrial tissue, was followed by primary closure of the atrial wall with a 4-0 suture. The paraganglioma near the left main coronary artery could be dissected safely on arrested heart, and the arterial supply was clipped.

The paraganglioma in the right atrioventricular groove was adjacent to the right coronary artery, of which large atrial and ventricular branches were carefully dissected and spared. Feeding branches of the paraganglioma were clipped. The tumour did not infiltrate the atrioventricular wall. The patient was extubated in the operating room and discharged on postoperative day 5 after an uneventful postoperative course.

**Figure 2:**
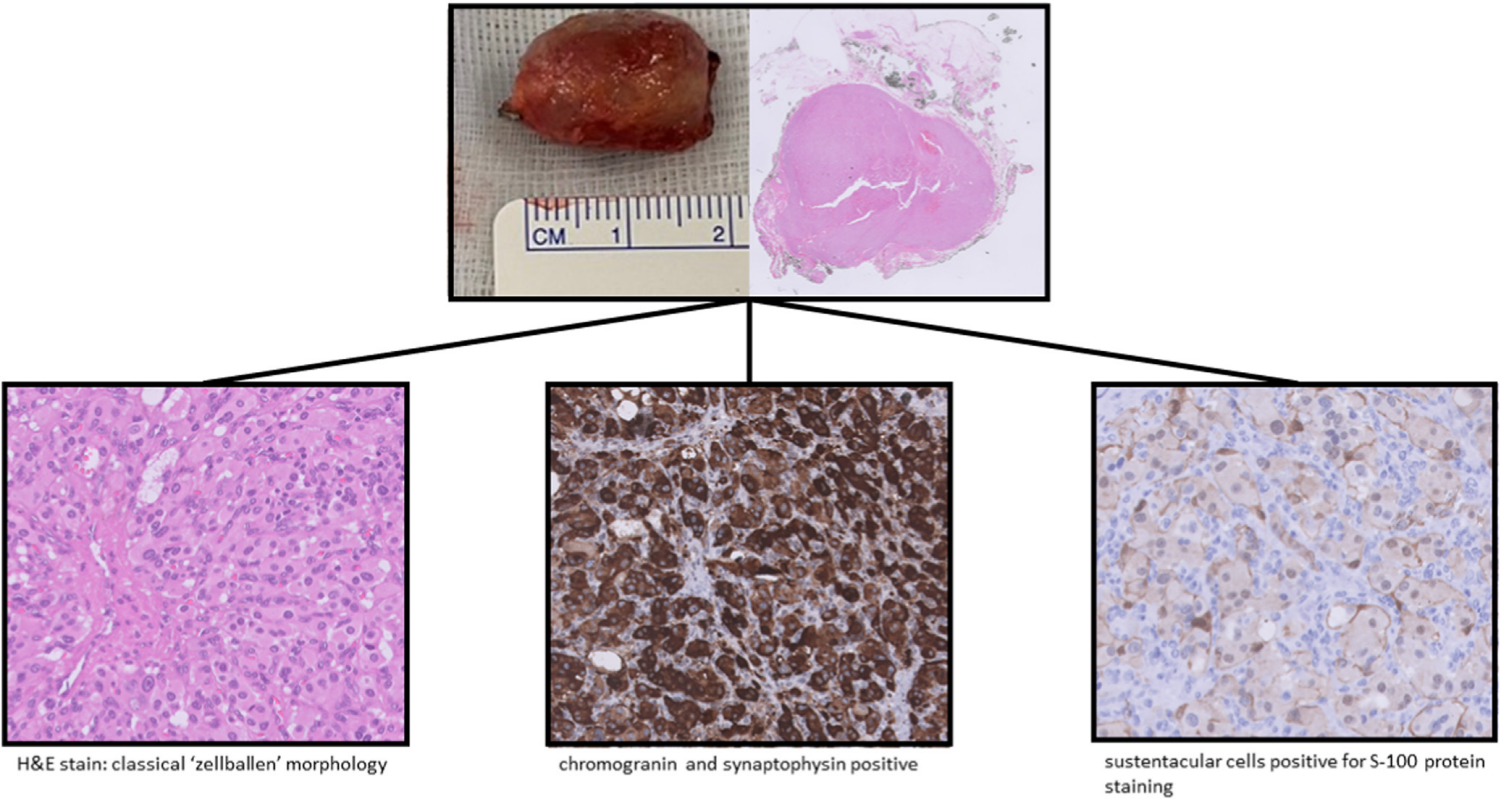
Histologic examination of the paragangliomas showed a typical Zellballen pattern. Chromogranin and synaptophysin were strongly positive, and sustentacular cells were positive for S-100 protein staining. H&E, hematoxylin and eosin.

Histologic examination of the excised tissue showed small nests (“*Zellballen*,” which is a German word for "ball of cells" pattern). Chromogranin and synaptophysin were strongly positive, and sustentacular cells were positive for S-100 protein staining (Fig. 2). All paragangliomas were resected in toto.

Repeated chest CT scan 5 months after surgery did not show any recurrence of pargangliomas. At latest follow-up 1 year after surgery, patient was still asymptomatic and in excellent clinical condition.

## Discussion

Since the first report of successful surgical resection of a cardiac paraganglioma in 1974, few studies have been published, mainly consisting of small case series and reports.^1^^,^^4^^,^^5^ Most cardiac paragangliomas are localized near the left atrium and the pulmonary veins.^1^^,^^5^ As data on surgical management of cardiac paragangliomas are scarce, different pre- and intraoperative management techniques have been reported.^1^ Here, we report a unique case of a patient with 4 paragangliomas, 2 of which were adjacent to the right and left coronary arteries. In contrast to previous reports with preoperative coiling or covered stenting of the supplying arterial branch, we demonstrate that direct surgical removal is feasible and safe.^1^^,^^3^

To improve and standardize the treatment of this rare disease, sharing worldwide experience with pre- and intraoperative management is important. Cardiac paragangliomas are often situated at critical locations (eg, near coronary arteries and the great vessels) and may infiltrate cardiac tissue. Preoperative electrocardiogram, triggered CT scan and coronary angiography is essential to determine whether surgical resection is feasible and to plan surgery. In addition, we recommend preoperative biochemical testing and chemical suppression, if necessary. Intraoperative planning for eventual concomitant coronary bypass grafting should always be undertaken. The benefit of preoperative covered stenting of coronary arteries is controversial, and these patients may require a reintervention at follow-up. In our opinion, the use of cardiopulmonary bypass facilitates identification and dissection of cardiac paragangliomas. When tumours are adjacent to the coronary arteries, cardioplegic arrest is recommended to facilitate resection and, if needed, concomitant coronary artery bypass grafting.

The presented case demonstrates that resection of cardiac paragangliomas is feasible and does not require preoperative interventions such as coiling or covered stenting of coronary arteries. On a cardioplegic heart, precise dissection and sparing of coronary artery branches can be accomplished. Postoperative lifelong surveillance is recommended to diagnose recurrent paragangliomas in a timely manner.

**Novel Teaching Points**

• In contrast to previous reports with preoperative coiling or covered stenting of the supplying arterial branch, we demonstrate that direct surgical removal of cardiac paragangliomas is feasible and safe. Coronary angiography is recommended to visualize vascularization of the paraganglioma.
• Bicaval venous cannulation is preferred in cases with possible invasiveness of right-sided tumours in cardiac tissue. Cardioplegic arrest is recommended to facilitate resection of paragangliomas adjacent to the coronary arteries.
